# A commentary on “Differentiation of pluripotent stem cells into striatal projection neurons: a pure MSN fate may not be sufficient”

**DOI:** 10.3389/fncel.2015.00177

**Published:** 2015-05-12

**Authors:** Berardino Porfirio, Annamaria Morelli, Renato Conti, Gabriella B. Vannelli, Pasquale Gallina

**Affiliations:** ^1^Department of Experimental and Clinical Biomedical Sciences “Mario Serio”, University of FlorenceFlorence, Italy; ^2^Department of Clinical and Experimental Medicine, University of FlorenceFlorence, Italy; ^3^Department of Surgery and Translational Medicine, University of FlorenceFlorence, Italy

**Keywords:** DARPP-32 neurons, embryonic stem cell striatal-committed progenitors, fetal striatal transplantation, Huntington's disease, striatal anlagen

Proof-of-concept has long been gained from both Huntington's disease (HD) animal models and pilot clinical trials that transplantation of fetal striatal tissue has the potential to offer a substitutive therapy to HD patients (Peschanski et al., 1995; Bachoud-Lévi et al., 2006; Reuter et al., 2008; Paganini et al., 2014). Nonetheless, in the stem cell era, the body of knowledge so far obtained from fetal tissue as cell source may well be handed over to the clinical exploitation of neural stem cells (Tabar and Studer, 2014).

Loss of DARPP-32 medium-sized spiny projection neurons (MSN) in the striatum is a hallmark of HD. Hence, production of this cell type from pluripotent stem cells holds promises for achieving brain repair (Dunnett and Rosser, 2014). Reddington et al. (2014) analyzed the shortcomings of targeting at just the MSN lineage and correctly pointed out that one has to readdress the question “what constitutes a functional striatal graft?” Important insights are in fact emerging from studies encompassing fetal striatal grafting and normal striatal development.

Caudate-putaminal transplantation of human striatal primordium is straightforward in its procedure, since just involves the injection of a dissociated cell suspension into the adult HD brain without any additional growth factors or other supply (Bachoud-Lévi and Perrier, 2014). Four reports provided long-term analysis of Unified HD Rating Scale performances after transplantation of a limited number of patients (Bachoud-Lévi et al., 2006; Reuter et al., 2008; Barker et al., 2013; Paganini et al., 2014). The results of these not-randomized studies are reportedly at variance. While Barker et al. (2013) found no sustained functional benefit due to deadly and/or insufficient number of grafted cells, the other three studies demonstrated some clinical benefit which paralleled with graft survival, development and function. In the Florence experience, some of the grafts have been growing for 9–12 months, then stopped, perhaps according to a self-limiting and time-scheduled pattern (Gallina et al., 2010, 2014; Mascalchi et al., 2014). In particular, Gallina et al. (2014) reported an illustrative case where the characterization of the intrinsic, multifaceted molecular asset of the graft was associated with its ability to perform those developmental steps that led to a viable structure remodeling basal ganglia anatomy. *In vitro* studies, aimed at characterizing the fetal striatal source used in transplantation protocols, revealed that human striatal precursor (HSP) cells isolated from 9 to 12-week-old human fetuses, possess the machinery for long-term survival, proliferation and differentiation (Sarchielli et al., 2014; Ambrosini et al., 2015). Indeed, HSP cells featured a mixed population of immature elements, neuronal/glial-restricted progenitors and striatal neurons, pointing to a plastic phenotype already committed to become striatum. This heterogeneous composition reflects that of striatal primordium and favors its regenerative potential in HD patients. In addition, HSP cells are well equipped for adaptation and survival to hypoxia (Ambrosini et al., 2015), one of the micro-environmental stress to which grafted cells are exposed when transplanted into the diseased host brain, where the loss of neurons is also accompanied with reduced trophic support due to both astrocyte and blood vessel atrophy (Cisbani et al., 2013). Further investigations on the mechanisms underlying normal striatal ontogenesis are needed to identify the optimal fetal source and the adequate developmental window in order to optimize protocols for the use of human fetal striatal transplantation therapy in HD. In this regard, both *in vitro* modeling and *ex vivo* experiments have recently provided a molecular definition of developing striatal anatomy, showing how transcriptional and functional processes converge to specify human striatal and neocortical neurons during development (Onorati et al., 2014). In particular, the observation that DARPP-32 is expressed in the human LGE together with other striatal markers, but also in the human cortical plate at 8–11 weeks (Onorati et al., 2014), should be considered when monitoring *in vitro* the differentiation of human pluripotent stem cells toward MSN, as well as when grafting fetal striatum.

Even if deeper understanding is needed to fully answer the question “what constitutes a functional striatal graft,” provided it is taken within the appropriate developmental stage, striatal primordium seems to fulfill the requirements for effective repair. We definitely agree that a pure MSN fate may not be sufficient for successful stem-cell based transplantation protocols, especially because multiple types of striatal neurons and glial cells are required for a full striatal reconstruction. Therefore, more sophisticated differentiation protocols will be necessary. In the meanwhile, it would be extremely important for people who are now living the dramatic condition of HD prospective trials be undertaken to assess the clinical utility of fetal-tissue based therapies. Certainly, several challenges remain to be faced, including overall optimization of graft procedure and patient management (Baizabal-Carvallo, 2014; Bachoud-Lévi and Perrier, 2014). However, based on what we have learned up to now, it seems appropriate not to neglect this approach and keep going. We owe it to patients.

## Conflict of interest statement

The authors declare that the research was conducted in the absence of any commercial or financial relationships that could be construed as a potential conflict of interest.
